# Case Report: KIF5B-ALK–rearranged renal cell carcinoma

**DOI:** 10.3389/fonc.2026.1825447

**Published:** 2026-06-15

**Authors:** Jiaxi Yao, Zhifei Xie, Guangyao Shi, Ming Tan, Zhuo Liu, Min Lu, Hongxian Zhang

**Affiliations:** 1 Department of Urology, Zhangye People’s Hospital Affiliated to Hexi University, Zhangye, China; 2 Institute of Urology, Hexi University, Zhangye, China; 3 Department of Urology, Wuqing District Second People’s Hospital, Tianjin, China; 4 Department of Urology, Chengwu County People’s Hospital, Heze, China; 5 Department of Urology, Peking University Third Hospital, Beijing, China; 6 Department of Pathology, Peking University Third Hospital, Beijing, China

**Keywords:** anaplastic lymphoma kinase, case report, KIF5B, rare, renal cell carcinoma

## Abstract

**Background:**

Anaplastic lymphoma kinase (ALK)–rearranged renal cell carcinoma (ALK-RCC) is a rare provisional entity characterized by fusion of the ALK gene with various fusion partner genes, resulting in oncogenic activation.

**Case presentation:**

We report a case of a 39-year-old Chinese man with ALK-RCC who underwent partial nephrectomy for a left renal tumor measuring 2.8 × 2.7× 2.6 cm. Histopathological examination revealed a renal epithelial tumor with atypical morphology. Immunohistochemistry demonstrated the occurrence of renal epithelial differentiation and excluded other entities. Next-generation sequencing confirmed a KIF5B-ALK gene fusion.

**Conclusion:**

This case presents the clinical data, morphology, immunohistochemistry, and molecular characteristics of a rare subtype of ALK–rearranged renal cell carcinoma with KIF5B-ALK fusion. The findings may enhance the clinical and pathological understanding of ALK-RCC among clinicians and pathologists. Managing ALK-RCC is challenging due to its rarity and limited treatment options. Targeted therapies directed at the ALK gene may have a role in patients with ALK-RCC.

## 1 Introduction

Over the past few decades, advances in molecular pathology and genetics have enabled the continuous identification of new potential subtypes of renal cell carcinoma (RCC). In 2022, the World Health Organization Classification of Tumors of the Urinary System and Male Genital Organs recognized anaplastic lymphoma kinase (ALK)–rearranged renal cell carcinoma (ALK-RCC) as a “tentative disease,” further clarifying the classification of renal cancer based on molecular markers (1). The clinical manifestations and histological characteristics of patients with ALK-RCC have been preliminarily recognized; however, these are mainly case reports and case series reviews.

ALK is a receptor tyrosine kinase belonging to the insulin receptor superfamily (2). It is encoded by the ALK gene located on chromosome 2p23 and consists of 1620 amino acids (3). ALK can fuse with various protein molecules, leading to its abnormal activation and promoting the activation of downstream signaling pathways, resulting in tumor proliferation and progression. ALK rearrangements have been reported in various malignant tumors, including inflammatory myoblastoma, neuroblastoma, diffuse large B-cell lymphoma, esophageal squamous cell carcinoma, breast cancer, anaplastic large cell lymphoma, and non-small cell lung cancer (4–7).

ALK-RCC is a rare subtype of renal epithelial tumor, accounting for 0.3% of all RCC cases. In 2011, Debelenko et al. reported that African American patients with sickle cell characteristics had renal cancer with VCL-ALK fusion (8). VCL encodes a vascular protein, which is a cytoskeletal protein related to cell-cell and cell-matrix connections and is one of several interacting proteins that anchor actin to the cell membrane. These carcinomas typically show unclear tumor boundaries, common chronic inflammatory cell infiltration (mainly lymphoplasmacytic infiltration), polygonal tumor cells, rich eosinophilic cytoplasm, clear cytoplasmic vacuolation, and sickle red blood cells in or outside the blood vessels, which may be partially resemble renal medullary carcinoma and/or collecting duct carcinoma. In children, ALK-rearranged RCC may resemble renal medullary carcinoma, showing medullary-centered, diffuse infiltrative growth with lymphoplasmacytic infiltrates, large polygonal discohesive or spindle cells, cytoplasmic vacuoles, and vesicular nuclei. In adults, tumors show heterogeneous solid architecture with mucinous cribriform, signet ring, and solid rhabdoid patterns, as well as high-grade eosinophilic cells with intracytoplasmic lumina. Overall, these tumors are architecturally heterogeneous eosinophilic neoplasms composed of polygonal, rhabdoid, signet ring, and spindle cells with mucin production and cytoplasmic vacuolation.

ALK-rearranged RCCs are known to have a variety of fusion partners including VCL, TPM3, EML4, STRN, CLIP1, KIF5B, KIAA1217, and HOOK1 (9). Most RCCs occur in adult patients without sickle cell characteristics and are therefore referred to as non-VCL-ALK RCC. KIF5B, a core member of the motor protein family, is a microtubule-dependent molecular motor protein that mediates intracellular anterograde substance transport of organelles and macromolecules such as mitochondria, vesicles, and signaling molecules, maintaining cell structure and signal homeostasis (10). When KIF5B-ALK gene fusion occurs, the ALK kinase domain is continuously abnormally activated, which drives tumor occurrence and progression through the excessive activation of downstream signaling pathways.

The fusion partner genes of non-VCL-ALK fusion-related RCCs and their correlation with histology have not yet been established. RNA sequencing may help to identify partner genes related to ALK fusion. Here, we report a rare case of renal cell carcinoma harboring a KIF5B-ALK fusion identified by next-generation sequencing (NGS).

## 2 Case description

A 39-year-old male patient was found to have a space-occupying lesion in the left kidney during a physical examination. The patient had no history of sickle cell disease or other significant medical conditions. He was admitted to Peking University Third Hospital on September 4, 2025, where he underwent enhanced abdominal computed tomography and plain magnetic resonance imaging.

Computed tomography imaging revealed a round iso-dense mass in the upper pole of the left kidney, measuring 2.8 × 2.7 × 2.6 cm. Contrast enhancement showed a slightly lower degree of enhancement than normal renal parenchyma. The initial diagnosis was a left-kidney mass. Magnetic resonance imaging revealed a nodular long T1 signal in the upper pole of the left kidney, with a slightly shorter T2 signal and patchy short T2 signal. Diffusion-weighted imaging demonstrated patchy high signal, with a reduced apparent diffusion coefficient value. The lesion had a clear boundary and measured approximately 2.9 × 2.8 × 2.6 cm (**Figure 1**).

**Figure 1:**
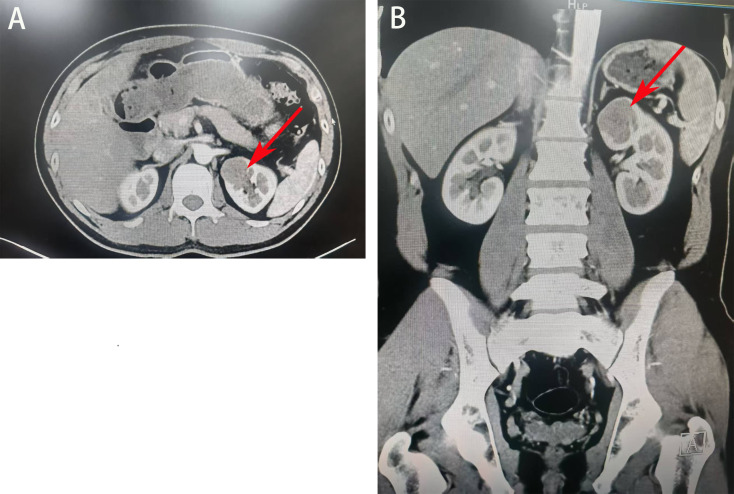
Computed tomography imaging shows a space-occupying lesion in the left kidney (red arrow). **(A)** Axial view, **(B)** Coronal view.

On September 8, 2025, the patient underwent posterior abdominal laparoscopic partial resection of the left kidney. During surgery, the tumor was located in the upper pole of the left kidney with a smooth surface. The tumor was completely removed without intraoperative complications (**Figure 2**). The visible part of the resected kidney tissue was 4.5 × 3.5 × 3 cm in size. The cut surface was gray-white and solid, without bleeding or necrosis. A focal gray-white scar was observed near the tumor, close to the capsule and 0.5 cm from the resection margin. The tumor did not invade the renal pelvis or calices.

**Figure 2:**
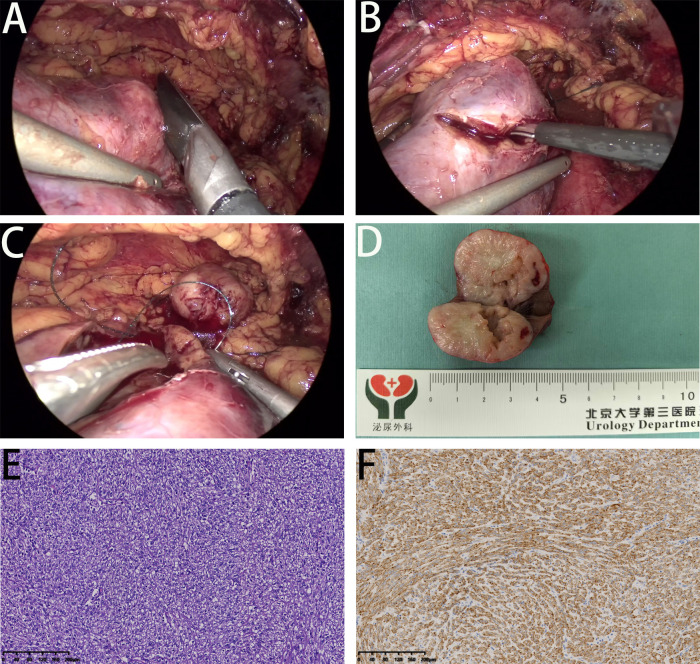
Surgical procedure and pathological results. **(A)** Localization of the tumor by ultrasound, **(B)** Removal of the tumor, **(C)** Closing of the wound, **(D)** Tumor specimen, **(E)** Hematoxylin and eosin staining, **(F)** ALK immunohistochemical staining. Original magnification: ×200.

Immunohistochemistry (IHC) excluded Perivascular Epithelioid Cell tumor (PEComa), CK mixed (+), PAX-8 (+), HMB45 (−), Cathepsin K (−), S-100 (−), SMA (−), TFE3 (−), SMMHC (−), Ki-67 (hot zone 15%+, average 5%), MiTF (−), and Melan-A (−) (**Figure 2**). It was recommended to perform tumor and peripheral blood NGS testing; subsequently, the patient underwent NGS testing (Illumina sequencer). The test covered the exons of 70 genes (some genes were covered in the full coding region, and some in the hotspot coding region) of fusion-related introns and variable splicing regions, totaling approximately 0.242M base pairs. The results revealed that the 24th exon of the KIF5B gene fused with the 20th exon of the ALK gene, confirming the presence of a ALK fusion/rearrangement (KIF5B-ALK) (E24:E20).The patient’s follow-up status one month after the surgery was good, with no tumor recurrence or metastasis.

## 3 Discussion

ALK-RCC accounts for less than 1% of all renal tumors (11). However, with the rapid development of molecular detection technologies and NGS, an increasing number of patients with ALK-RCC have been identified. In the present case, a 39-year-old young male patient had an intrinsic tumor in the T1a stage, which was detected at an early stage and without metastasis. Laparoscopic surgery was performed and good therapeutic effects were achieved.

The pathological specimen of the patient presented a grayish-white appearance, similar to sarcoma manifestations and different from classic clear cell carcinoma. Generally, ALK-RCC is an isolated tumor that is not usually accompanied by other syndromes. The gross pathological features typically indicated solid or cystic solid tumors originating from the medulla or cortex. After the incision, the sections primarily appeared white and gray to yellow.

The most common translocation in patients with ALK RCC is VCL-ALK, particularly in young African patients with sickle cell characteristics. To date, no recurrence or distant metastasis has been reported in children with VCL-ALK RCC. ALK copy number gain can be identified in up to 10% of clear cell RCCs and is associated with worse cancer-specific survival.TPM3-ALK and EML4-ALK fusions consist mainly of papillary, solid, and mucinous sieve-like components. STRN-ALK translocation is typically characterized by papillary, solid, tubular, mucinous sieve-like structures, and sand-like bodies. A retrospective study by Sukov et al. analyzed 534 patients and found that two patients had ALK rearrangements (12). Similarly, Sugawara et al. found two cases of ALK rearrangements among 355 Japanese patients with renal tumors (13). Other studies from Turkey, Korea, and China have reported similarly low detection rates, generally below 1% (14–16). Conversely, Gorczynski et al. analyzed the results of 1019 cases of renal cell carcinoma and found no positive cases of ALK-RCC (<1%) (17). Notably, Arizpe et al. reported five cases of ALK-RCC with ARNT, EML4, NUMA1, TPM3, and VCL fusions (18), whereas Tu et al. reported the case of a 30-year-old male with RCC with ALK-TPM3 gene fusion and ALK amplification (19).

Currently, no large-scale phase III clinical studies have confirmed ALK-RCC treatment. The discovery of ALK-positive tumors has promoted the development of targeted drug therapies using ALK inhibitors (ALK-i). Crizotinib was the first ALK-i drug approved for the treatment of metastatic non-small cell lung cancer (20). Subsequently, second-generation ALK-is (alectinib, brigatinib, and ceritinib) and third-generation ALK-is (lorlatinib) were launched in the United States (21, 22). All of these drugs have demonstrated undeniable efficacy in patients with advanced non-small cell lung cancer, completely changing the treatment outcome for ALK-positive tumors. Pal et al. reported that three patients with advanced papillary RCC treated with second-generation alectinib achieved partial remission (23). Zhou et al. reported that a young woman with local renal cell carcinoma who developed lung metastasis after nephrectomy was treated with sunitinib as first-line therapy. After IHC and fluorescence *in situ* hybridization testing showed ALK positivity, the patient was switched to crizotinib for systemic treatment and achieved stable disease (24). Varchetta et al. reported a case of a patient with recurrent renal cancer who tested positive for ALK-RCC and received alectinib, achieving partial remission (25). Thorner et al. reported the case of a 12-year-old patient with recurrent ALK-RCC who received ALK-i treatment and was still receiving treatment (26).

With the positive therapeutic effects of immunotherapy in various tumor fields, there have also been some attempts to treat ALK-RCC. Zhang et al. reported three patients with TPM3, VCL, and EML4 fusion ALK-RCC and found that patients with immune-excluded phenotypes may not benefit from immunotherapy (27). Although there are few studies on the treatment of ALK-RCC with ALK-i, all patients with ALK-RCC who received ALK-i treatment reported in the literature achieved treatment responses; however, this is only a report of a few cases and still requires large-scale clinical studies for confirmation. The identification of ALK rearrangements in tumor types has subsequently gained importance owing to the availability of ALK inhibitor-targeted therapies.

The age range of patients with ALK-RCC is wide, and the tumor morphology varies. VCL-ALK RCCs generally have sickle cell-like traits, with cell morphology characterized by the formation of solid vacuolated cytoplasmic patches of polygonal cells. In contrast, non-VCL-ALK RCCs have a wide age range and a diverse morphological spectrum, including papillary, tubular, cribriform, and popcorn-like patterns. The patient in the present case had KIF5B-ALK RCC, a rare ALK fusion gene. The present case of KIF5B-ALK-rearranged renal cell carcinoma exhibited a gray-white solid cut surface, polygonal tumor cells with eosinophilic cytoplasm, and no sickle cell trait, which could be easily confused with several other renal entities. The main differential diagnoses include: (1) Renal medullary carcinoma typically occurs in young patients with sickle cell disease or trait, shows infiltrative growth, reticular or microcystic pattern, and prominent lymphoplasmacytic infiltration; however, ALK-RCC (especially VCL-ALK) can mimic it closely. In such cases, ALK IHC is essential because renal medullary carcinoma is negative for ALK, whereas positive staining strongly supports ALK-RCC. (2) Collecting duct carcinoma-presents with infiltrative borders, desmoplastic stroma, high−grade eosinophilic or hobnail cells, and no sickle cell background. Its morphology overlaps with some ALK-RCCs, but collecting duct carcinoma lacks ALK rearrangement. Thus, ALK testing helps to distinguish a potentially targetable tumor from an aggressive carcinoma with limited treatment options. (3) Papillary renal cell carcinoma (type 2) -can show pseudostratified nuclei, eosinophilic cytoplasm, and foamy macrophages. Non−VCL-ALK fusions (e.g., TPM3−ALK, EML4-ALK) may exhibit papillary components. ALK IHC is not routinely performed for papillary RCC, but when morphology is atypical (e.g., solid areas, rhabdoid cells, or mucin production), ALK testing should be considered to avoid missing an ALK-rearranged subtype. (4) MiT family translocation RCC (Xp11.2 or t(6;11))-often occurs in young patients, shows nests of clear to eosinophilic cells with psammoma bodies, and expresses TFE3 or TFEB. However, ALK-RCC may also present with clear cell morphology and rarely express TFE3. ALK IHC and TFE3/TFEB IHC combined with molecular studies (FISH or NGS) are necessary for accurate subclassification, as the clinical behavior and potential for ALK-inhibitor therapy differ markedly. (5) Mucinous tubular and spindle cell carcinoma (MTSCC) features bland spindle cells, low−grade nuclei, and mucinous stroma, typically negative for ALK. If a tumor shows overlapping features with ALK-RCC (e.g., spindle cells in the present case were not prominent but could be seen in other ALK-RCCs), ALK IHC can rapidly exclude ALK-rearranged RCC. (6) Rhabdoid RCC or clear cell RCC with rhabdoid features-demonstrates discohesive cells with eosinophilic cytoplasmic inclusions, high Ki-67, and often aggressive behavior. In summary, for any renal tumor with atypical, heterogeneous, or hybrid morphology that cannot be confidently classified into a standard RCC subtype after routine IHC, ALK IHC should be performed as a gatekeeping screening tool, followed by confirmatory molecular testing. This approach not only prevents misdiagnosis but also uncovers a therapeutically actionable target.

ALK IHC has proven to be an important screening tool for diagnosing ALK-positive cell lung cancer, and ALK-positive patients are suitable for treatment with ALK inhibitors. Ventana-D5F3 IHC can be performed quickly to determine whether one can benefit from ALK inhibitors. Fluorescence *in situ* hybridization is also a standard method for detecting ALK rearrangements, but it cannot detect unknown ALK fusion partners. We advocate a routine ALK IHC screening for “unclassifiable RCCs” with heterogeneous features.

Advantages and limitations of ALK immunohistochemistry in the diagnosis of ALK-RCC. ALK IHC, particularly with the Ventana D5F3 antibody, serves as a rapid and cost-effective screening tool for identifying potential ALK-rearranged renal cell carcinomas, especially in tumors with unclassifiable or heterogeneous morphology. Its main advantage is the ability to triage cases for confirmatory molecular testing, as a strong and diffuse cytoplasmic or dot−like staining pattern correlates well with the presence of ALK fusions. However, ALK IHC in RCC has several limitations. First, false-negative results can occur in certain fusion types (e.g., VCL−ALK may show weaker or focal staining), and the staining intensity may vary with different fusion partners. Second, false−positive cytoplasmic staining has been reported in some RCC subtypes (e.g., high-grade clear cell RCC or MiT family translocation RCC) due to non−specific cross−reactivity or ALK copy−number gains without rearrangement. Third, ALK IHC does not identify the specific fusion partner, which is increasingly relevant as different partners might influence prognosis or therapeutic response. Important practical considerations include: (i) using a validated antibody clone and automated platform; (ii) interpreting only unequivocal, strong cytoplasmic or dot−like positivity as suspicious for ALK rearrangement; (iii) always confirming positive IHC results with a molecular method such as FISH, RT-PCR or next−generation sequencing; and (iv) being aware that ALK IHC is not a substitute for molecular testing in cases with atypical morphology but negative IHC, as rare fusions may escape detection by current antibody clones. Therefore, while ALK IHC is a valuable initial screening tool for ALK−RCC, its results should be interpreted with caution and validated by molecular techniques to avoid misdiagnosis. RT-PCR and NGS can be used to precisely detect specific fusion genes. Although the clear identification of fusion genes cannot provide further benefits for treatment at present, it indicates the direction for precise gene therapy in the future.

A diagnostic pitfall that merits attention is the possibility of misinterpreting a metastatic ALK−rearranged tumor from other primary sites (e.g., lung cancer, anaplastic large cell lymphoma, or inflammatory myofibroblastic tumor) as a primary ALK-RCC, or conversely, dismissing a true primary ALK-RCC as a metastasis from an unknown extrarenal ALK-positive neoplasm. Both scenarios share similar ALK IHC positivity and may even involve overlapping fusion partners such as EML4-ALK or KIF5B−ALK. To avoid this pitfall, a thorough clinical history, imaging workup to exclude extra−renal primary tumors, and a panel of organ-specific immunohistochemical markers (e.g., PAX-8, PAX-2, CK7, CD10, etc.) are essential. In the present case, the patient had no prior or concurrent ALK−positive tumors elsewhere, and the tumor strongly expressed PAX-8 and CK while lacking markers of other lineages, confirming its primary renal origin. Therefore, when encountering an ALK-positive renal tumor, a systematic approach integrating clinical, imaging, immunohistochemical, and molecular data is crucial to distinguish primary ALK-RCC from a metastatic mimic.

Owing to the rarity and morphological heterogeneity of ALK-RCC, diagnosis is extremely difficult. There are frequent misdiagnoses and missed diagnoses, such as misdiagnosis as renal medullary carcinoma, collecting duct RCC, papillary RCC, MiT family translocation RCC, rhabdoid RCC (or transparent cells with rhabdoid features), and mucinous tubular and spindle cell carcinoma. From a histopathological perspective, ALK-RCC is defined by heterogeneous and mixed morphology and has a wide differential diagnosis including fumarate hydratase (FH)-deficient RCC, SMARCB1-deficient renal medullary carcinoma, papillary RCC, MiTF family translocation RCC (specifically, Xp11.2 translocation RCC and TFEB translocation RCC), mucinous tubular and spindle-cell carcinoma (MTSCC), rhabdoid RCC (transparent cells with rhabdoid features) (28). Improving diagnostic accuracy and screening efficiency requires several future efforts. First, prospective application of routine ALK immunohistochemistry in “unclassifiable” or morphologically challenging RCCs should be encouraged, with integration of artificial intelligence−assisted digital pathology to recognize subtle histologic patterns predictive of ALK fusions. Second, NGS panels that cover a wide range of fusion partners (including rare ones such as KIF5B, CLIP1, and KIAA1217) should become more accessible in routine practice, preferably by RNA−based NGS to detect both canonical and novel fusions. Third, liquid biopsy approaches, particularly circulating tumor DNA or RNA sequencing, may emerge as non−invasive screening tools for detecting ALK rearrangements in patients with advanced or metastatic disease when tissue is limited. Fourth, establishment of multi−center registries and international collaborations will help define the natural history, genotype−phenotype correlations, and optimal screening algorithms for ALK−RCC. Finally, as ALK inhibitors become increasingly available, developing rapid, point−of−care molecular assays (e.g., digital PCR) could enable timely screening of high−risk patients or those with relapsed disease. These directions will not only reduce underdiagnosis but also facilitate precision medicine for this rare but targetable renal cancer subtype.

For rare non−VCL ALK−RCCs (e.g., KIF5B−ALK, CLIP1−ALK, KIAA1217−ALK). First, these tumors often lack the classic sickle−cell background and medullary location seen in VCL−ALK RCC; instead, they present as circumscribed cortical or medullary masses in young to middle−aged adults without hemoglobinopathy. Second, morphology is highly variable but frequently shows mixed patterns (papillary, solid, cribriform, or mucinous areas) with eosinophilic cells containing cytoplasmic vacuoles or intracytoplasmic lumina, and occasionally spindle or rhabdoid cells. Third, ALK IHC (Ventana D5F3) typically shows strong, diffuse cytoplasmic or dot−like positivity, but weaker or focal staining can occur; therefore, any unequivocal ALK positivity, even if focal, in an unclassifiable RCC should trigger molecular confirmation. Fourth, because some rare fusion partners do not reliably produce a strong IHC signal, a negative ALK IHC does not entirely exclude ALK−RCC – if clinical or morphological suspicion remains high, proceed directly to RNA−based NGS. Many primary hospitals do not conduct NGS monitoring; therefore, complex and special types of renal cancers are more likely to be overlooked. ALK-RCC incidence may have been underestimated because of the lack of routine detection methods. Therefore, for patients with complex morphology that requires differential diagnosis and is difficult to clearly identify, necessary ALK testing should be performed. NGS testing can better assist in diagnosis, prevent misdiagnosis, and guide patients for appropriate treatment (29).

## 4 Patient perspective

The patient was diagnosed with renal cell carcinoma harboring KIF5B-ALK gene fusion. Prior to treatment, the patient exhibited anxiety regarding the diagnosis and clinical prognosis. Following partial nephrectomy, the patient recovered well postoperatively. At the one-month follow-up, no evidence of tumor recurrence or metastasis was detected. The patient was satisfied with the surgical outcome and overall treatment process, and maintained a positive outlook for subsequent follow-up and daily life.

## Data Availability

The raw data of this article will be made available by the corresponding authors, without undue reservation.
